# LegionProfiler: a computational tool for the identification of virulence factors and classification of *Legionella pneumophila* serogroup 1 isolates

**DOI:** 10.1093/bioinformatics/btaf398

**Published:** 2025-07-12

**Authors:** Oluwafemi A Sarumi, Wilhelm Bertrams, Oliver Schwengers, Jan-Paul Herrmann, Torsten Hain, Laurine Kieper, Markus Petzold, Alexander Goesmann, Bernd T Schmeck, Dominik Heider

**Affiliations:** Institute of Medical Informatics, University of Münster, Albert-Schweitzer-Campus, Münster 48149, Germany; Institute for Lung Research, Universities of Giessen and Marburg Lung Center (UGMLC), Philipps-Universität Marburg, Marburg 35043, Germany; Core Facility Flow Cytometry – Bacterial Vesicles, Philipps-University Marburg, Marburg 35043, Germany; Bioinformatics and Systems Biology, Justus Liebig University Giessen, Giessen 35392, Germany; Institute of Medical Microbiology, Justus Liebig University Giessen, Giessen 35392, Germany; Institute of Medical Microbiology, Justus Liebig University Giessen, Giessen 35392, Germany; Institute for Medical Microbiology and Virology, Medical Faculty, and University Hospital Carl Gustav Carus, TUD Dresden University of Technology, Dresden 01307, Germany; Institute for Medical Microbiology and Virology, Medical Faculty, and University Hospital Carl Gustav Carus, TUD Dresden University of Technology, Dresden 01307, Germany; Bioinformatics and Systems Biology, Justus Liebig University Giessen, Giessen 35392, Germany; Institute for Lung Research, Universities of Giessen and Marburg Lung Center (UGMLC), Philipps-Universität Marburg, Marburg 35043, Germany; Core Facility Flow Cytometry – Bacterial Vesicles, Philipps-University Marburg, Marburg 35043, Germany; Institute of Medical Informatics, University of Münster, Albert-Schweitzer-Campus, Münster 48149, Germany

## Abstract

**Summary:**

*Legionella pneumophila* has significantly contributed to multiple cases of pneumonia with a high rate of mortality globally. Its ability to exploit host mechanisms through several expressed virulence factors poses challenges for diagnosis, treatment, and outbreak control. To address this, we developed LegionProfiler, a computational tool that swiftly identifies virulence factor protein domains within genome assemblies of *Legionella pneumophila* serogroup 1 isolates and classifies them into high- or low-virulence groups. LegionProfiler automates the probing of genome assemblies for virulence-associated protein domains and determines the isolate’s potential to cause severe pneumonia infection. The LegionProfiler workflow is made available through a user-friendly interface to enhance technical control of infectious sources and adds important insights to the general epidemiology of clinical isolates. It could also support the development of targeted therapeutic strategies that will improve patient treatment.

**Availability and implementation:**

LegionProfiler is freely accessible as a web service at https://legionprofiler.uni-muenster.de, and can also be run locally in a Docker container. The source code can be found at https://imigitlab.uni-muenster.de/heiderlab/legionprofiler or at Zenodo (DOI: 10.5281/zenodo.15592325).

## 1 Introduction

Pneumonia ranks as the fourth leading cause of death worldwide, with mortality rates remaining consistently high over the past 60 years. Despite significant advances in medicine, it remains a prominent cause of morbidity and mortality, especially in the elderly (Henig and Kaye 2017). Among the most common causes of severe pneumonia is *Legionella pneumophila* (*L. pneumophila*), with increasing prevalence in the EU (Cilloniz *et al.* 2016). The surveillance systems of Europe and the USA report an increase in the number of cases of Legionnaires’ disease, a serious pneumonia caused by legionella. The majority of cases can be attributed to *L. pneumophila* serogroup (Sg) 1, which represents approximately 85% of the Legionella disease cases (European Centre for Disease Prevention and Control 2021).

*Legionella pneumophila* (Monteiro *et al.* 2022) is a Gram-negative, non-encapsulated, aerobic bacterium with a single polar flagellum. It is an intracellular pathogen that can be found ubiquitously in freshwater sources, especially in man-made environments such as water distribution systems and cooling towers. *Legionella pneumophila* is highly adapted to intracellular replication, residing naturally in eukaryotes such as amoebae within aquatic environments. Human infection occurs through the inhalation of legionellae-contaminated aerosols, enabling the bacteria to infect alveolar macrophages and cause pneumonia. The complete genome sequencing of three clinical *L. pneumophila* isolates (Cazalet *et al.* 2004) paved the way for understanding its molecular biology, and subsequent comparative genomic studies of 180 Legionella strains revealed high genome plasticity and frequent horizontal gene transfer. Further investigation of the Legionella lifecycle shows that Legionella exhibit a biphasic life cycle and define transmissive and replicative traits according to gene expression profiles (Barbosa *et al.* 2024).

*L. pneumophila* expresses >300 virulence factors (Oliva *et al.* 2018) that can be injected into the cytosol of the host cell through a Type IV secretion system, manipulating key host cell processes such as vesicle trafficking and gene expression (Gomez-Valero *et al.* 2019). These virulence factors sometimes include eukaryote-like protein motifs, acquired during co-evolution with host organisms, which optimize bacterial survival (Gomez-Valero *et al.* 2011, Herbel *et al.* 2022). The genome of *L. pneumophila* encodes a significant number of these motifs, making their accurate annotation essential to understand disease mechanisms and identify potential therapeutic targets.

A comprehensive understanding of the interplay between legionellae and host immune cells is essential to address the clinical challenges posed by pneumonia. Although antimicrobials have shown promise, they alone are insufficient to address these challenges, emphasizing the need for additional and adjunctive treatment strategies and more efforts in genomic research. In this study, we introduce LegionProfiler, a computational pipeline for the fast identification of virulence factor protein domains in legionellae genomes and classification of clinical isolates as either high or low-virulence strains, thereby providing insights into their pathogenic potential. This could be used as a factor for the risk analysis of environmental sources as well as an additional factor for a personalized and targeted treatment.

## 2 Materials and methods

### 2.1 Dataset

The experimental dataset comprises 38 clinical isolates of *L. pneumophila* Sg 1 Table 1, available as supplementary data at *Bioinformatics* online, collected from outbreaks and categorized into two groups based on their mAb 3/1 epitope status (Helbig *et al.* 2002, Palusinska-Szysz *et al.* 2019, Wee *et al.* 2021). Among these 38 isolates, 14 were identified as mAb 3/1-positive (high-virulence) strains, while 24 were identified as mAb 3/1-negative (low-virulence) strains. The procedure for the isolation and characterization of *L. pnemunohila* Sg 1 isolates was described in the Text, available as supplementary data at *Bioinformatics* online. These isolates were sequenced using the Oxford Nanopore Technology (ONT) platform, which generated the raw sequence reads. These reads were preprocessed using Dorado https://github.com/nanoporetech/dorado for super high accuracy basecalling, trimming, and demultiplexing, and filtered with Filtlong https://github.com/rrwick/Filtlong, applying a length threshold of 1 kbp to ensure high-quality data. The filtered reads were then assembled with Flye (Kolmogorov *et al.* 2019), re-oriented using Dnaapler (Bouras *et al.* 2024), and polished with Medaka https://github.com/nanoporetech/medaka to improve the quality of the genome assemblies.

For the subsequent analysis, 275 published virulence factors (Burstein *et al.* 2009, Zhu *et al.* 2011) were curated and used for the sequence alignment process, thereby facilitating the identification of known virulence factors in genome assemblies. The identified virulence factors were then utilized to develop a predictive model using the Huber regressor statistical modeling method to enhance a seamless categorization of new isolates into high-virulence and low-virulence groups. Furthermore, an independent dataset was used to validate the performance of the predictive model. The validation dataset comprised genome assemblies of 39 Legionella isolates made up of 22 high virulence and 17 low virulence isolates, which were originally obtained from diverse respiratory samples of patients at the Legionella reference laboratory in Dresden, Germany. In contrast to the reference data, these validation isolates were sequenced using Illumina short-read technology, and the assembly was performed using the Ridom SeqSphere+ version 4.0 software (Jünemann *et al.* 2013).

### 2.2 Sequence alignment and annotation

We functionally annotated the isolates’ genome assemblies using the BLASTX algorithm (Camacho *et al.* 2009) that aligned them against a curated database comprising 275 protein sequences associated with known virulence functions. BLASTX translates the nucleotide sequences of the assemblies into amino acid sequences across all six possible reading frames, enabling the detection of potential protein-coding regions. The translated sequences were then aligned with the curated virulence factor protein database, offering valuable functional insights into the nucleotide sequences and aiding the discovery of potential virulence factor protein domains within the genome assemblies.

### 2.3 Feature extraction process

The feature extraction process involved refining the BLASTX output to facilitate the identification of relevant virulence factors with a high degree of confidence. Initially, false-positive factors were removed by implementing a filtering criterion based on an identity score threshold of 90%. Subsequently, the calculation of sequence coverage was computed to ascertain the proportion of each virulence factor’s sequence that was covered by the identified factors in the BLASTX output. Additionally, a second-level filtering step was applied, retaining only factors with a sequence coverage of at least 90%. This two-layered filtering step ensured that the extracted virulence factor protein domains in the genome assemblies were relevant and of high confidence.

### 2.4 Predictive model

**Figure 1.**
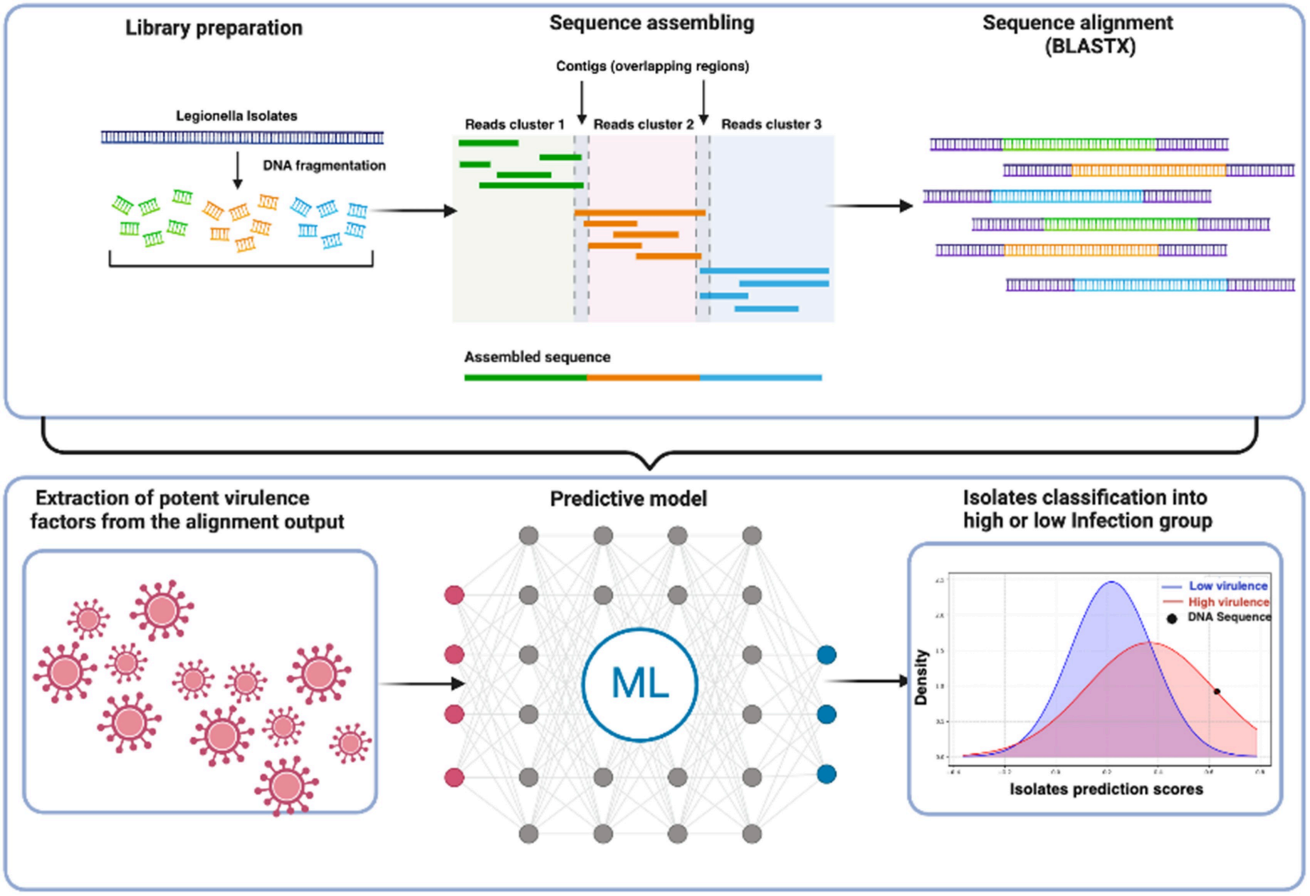
Visualization of the LegionProfiler workflow. Sequence alignment was performed on the input genome assemblies of the *L. pneumophila* Sg 1 isolates. High-confidence virulence factors were then extracted from the alignment results. Huber regressor was used to build a predictive model for the subsequent classification of isolates into high or low-virulence groups. Figure was created with Biorender.com

We opted for a regression model to build our predictive model due to the small size and imbalanced nature of our training dataset. In our preliminary experiments, alternative models, such as Random Forest, exhibited overfitting and performed poorly on the validation data. In contrast, the Huber regressor model offered better generalization and robustness to outliers by using a compact loss function. Thus, we used the Huber regressor model to develop a predictive model that effectively captures the relationship between the identified virulence factors in the Sg 1 isolates and their corresponding virulence groups as described in Fig. 1.

Given that $X=\{x_{1},x_{2},\ldots ,x_{n}\}$ is the set of input features representing the identified virulence factors in the isolates, and $\;y=\{y_{1},y_{2},\ldots ,y_{n}\}$ are their corresponding labels indicating infection outcomes, where $y_{i}\in \{0,1\}$ represents the binary classification of low or high virulence for each bacterial isolate. Then, we computed the Huber regressor model that uses a minimized loss function as defined in Eq. 1. The loss function transitions between quadratic loss (for small residuals) and linear loss (for large residuals).


(1) $$L_{\delta }(r)=\{\begin{array}{cc} \frac{1}{2}r^{2}, & \text{for}\;|r|\leq \delta \\ \delta (|r|-\frac{1}{2}\delta ), & \text{for}\;|r|>\delta \end{array}$$

where $r=y-\hat{y}$ is the residual, and δ is a threshold parameter that determines the transition between quadratic and linear loss, thus controlling the model’s sensitivity to outliers. The model optimizes the objective function *f* as shown in Equation (2).


(2) $$f=\arg\underset{\beta }{\min}\sum_{i=1}^{n}L_{\delta }(y_{i}-X_{i}\beta )$$

where β is the regression coefficient vector that determines the relationship between identified virulence factors and their corresponding virulence groups. Thus, the trained model generates predictions using Equation (3).


(3) $$\hat{y}=X\beta$$

where $\hat{y}$ is the estimated virulence probability. To classify isolates into high or low-virulence groups, a threshold θ is applied as shown in Equation (4).


(4) $${\hat{y}}_{i}=\{\begin{array}{cc} 1, & \text{if}\;{\hat{y}}_{i}>\theta \\ 0, & \text{otherwise} \end{array}$$

where θ is determined by selecting the optimal threshold from the ROC curve plots. The optimal threshold is selected as the point that is closest to the perfect classifier (0,1) using the Euclidean distance minimization approach as defined in Equation (5).


(5) $$\theta^{*}=\arg\underset{\theta }{\min}\sqrt{{\left(1-\text{TPR}(\theta )\right)}^{2}+{\left(\text{FPR}(\theta )\right)}^{2}}$$

where TPR is the true positive rate, and FPR is the false positive rate. This method ensures that the selected threshold maximizes true positives while minimizing false positives in the prediction outcomes.

## 3 Results

When developing our predictive model, we trained three regression models—Huber regression, Logistic regression, and Linear regression using the virulence factors extracted from 38 *L. pneumophila* Sg 1 clinical isolates. These models were then evaluated on our experimental validation set of 39 isolates to assess their predictive performance in classifying isolates as high or low infection risk. As shown in Table 1, Huber regression outperformed the other models, achieving the highest accuracy of 0.72 and MCC of 0.44. Also, the Huber model demonstrates strong specificity and moderate sensitivity, indicating its potential to classify isolates as high or low virulence accurately. From a public health perspective, high specificity is valuable for optimizing resources in low-resource settings by minimizing false alarms and unnecessary interventions. Nevertheless, higher sensitivity is desirable to ensure true cases are not missed. Also, to mitigate the effects of the FP and FN from the Huber model, we recommend that users interpret predictions in conjunction with other genomic or phenotypic data. Logistic regression demonstrated the weakest performance, with an accuracy of 0.54, MCC of 0.16, and a poor sensitivity of 0.32, indicating a significant wrong classification of positive cases. Linear regression exhibited an extremely high sensitivity of 1.0 but completely failed in specificity, misclassifying all negative cases as positive.

**Table 1.** Performance comparison of models in classifying *L. pneumophila* Sg 1 isolates as high or low risk strains based on: Matthews correlation coefficient (MCC), accuracy (ACC), sensitivity (Sen), specificity (Spe), true positives (TP), true negatives (TN), false positives (FP), and false negatives (FN).

Model	MCC	ACC	Sen	Spe	TP	TN	FP	FN
Huber-R	0.44	0.72	0.68	0.76	15	13	04	07
Linear-R	0.00	0.60	1.00	0.00	22	00	17	00
Logistic-R	0.16	0.54	0.32	0.82	07	14	03	15

## 4 Conclusion

LegionProfiler demonstrates efficient performance with a peak memory usage of 600 MB and an average runtime of 11.8 seconds, as shown in Fig. 1, available as supplementary data at *Bioinformatics* online when run locally in a Docker container. LegionProfiler is a reliable, open-source tool available as a web application with a user-friendly interface for seamless identification of virulence factors in *L. pneumophila* Sg 1 isolates, and classification of isolates as high or low virulence strains. By automating the detection of virulence-associated protein domains within genome assemblies, LegionProfiler aids in discovering the virulence potential of Sg 1 isolates with high accuracy. It reveals a correlation between an epidemiological marker on the bacterial surface (mAb type) and the type 4 secretion system substrate configuration, providing a composite score to assess a strain’s infectious potential. LegionProfiler has the potential to enhance isolates’ risk assessment and thereby the risk assessment of environmental sources. It also strengthens epidemiological surveillance by adding a new layer in the description of patient isolates, and supports the development of targeted therapeutic strategies for Legionellosis. By facilitating informed decision-making in both clinical and public health contexts, LegionProfiler will ultimately contribute to more effective management of Legionnaires’ disease.

## Supplementary Material

btaf398_Supplementary_Data

## Data Availability

The data underlying this article are available at https://imigitlab.uni-muenster.de/heiderlab/legionprofiler.
