# Supplementary material for: LegionProfiler: a computational tool for the identification of virulence factors and classification of Legionella pneumophila serogroup 1 isolates
Source: Bioinformatics. 2025 Jul 12;41(7):btaf398. doi: 10.1093/bioinformatics/btaf398 (PMC12304514; doi:10.1093/bioinformatics/btaf398)
Supplement: btaf398_Supplementary_Data [file btaf398_supplementary_data.pdf]

Supplementary Information for

LegionProfiler: A computational tool for the identification of virulence factors and classification of *Legionella pneumophila* serogroup 1 isolates

Oluwafemi A. Sarumi, Wilhelm Bertrams, Oliver Schwengers, Jan-Paul Herrmann, Torsten Hain, Laurine Kieper, Markus Petzold, Alexander Goesmann, Bernd T. Schmeck, Dominik Heider

Corresponding: Dominik Heider.
E-mail: dominik.heider@uni-uni-muenster.de

This PDF file includes:

Supplementary text
Table S1
Fig. S1

Supplementary Information Text

Isolation and characterization of *Legionella pneumophila* serogroup 1 isolates

The bacterial genomic DNA was extracted using the PureLink Genomic DNA Mini Kit (Thermo Fisher Scientific, Waltham, MA, USA) according to the manufacturer's recommendations. For DNA library preparation, Native Barcoding Kit SQK-NBD114.24 (Oxford Nanopore Technologies, Oxford, UK) and corresponding supplemental materials (as advised by the manufacturer) were applied. Actual genome sequencing of the libraries was conducted with MinION Mk1B device on MinION Flow Cell (FLO-MIN114,R10.4.1) as advised by Oxford Nanopore Technologies.

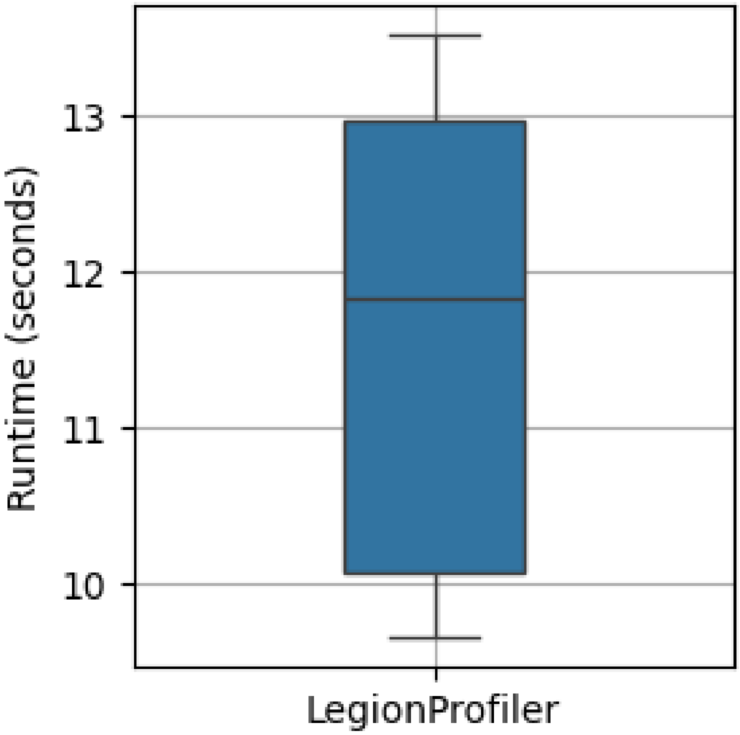

Fig. S1. Visualization showing the average runtime of LegionProfiler in 10 runs in a Docker container using isolates' sequences of varying sizes.

Table S1. Legionella isolates and their mAb types

SN	File_Name	Isolate	Group	mAb 3/1
1	MR_Legionella_1	Murcia	Philadelphia	Positive
2	MR_Legionella_2	Uppsala 3	Knoxville	Positive
3	MR_Legionella_3	L12-360	Allentown/France	Positive
4	MR_Legionella_4	L13-444	Knoxville	Positive
5	MR_Legionella_5	L13-435	Knoxville	Positive
6	MR_Legionella_6	W13-845-1	Knoxville	Positive
7	MR_Legionella_7	L08-336	Knoxville	Positive
8	MR_Legionella_8	L03-315	Knoxville	Positive
9	MR_Legionella_9	L20-240	Knoxville	Positive
10	MR_Legionella_11	L15-310	Benidorm	Positive
11	MR_Legionella_12	L10-071	Knoxville	Positive
12	MR_Legionella_13	Alcoy1999	Knoxville	Positive
13	MR_Legionella_17	L23-267	Allentown/France	Positive
14	MR_Legionella_18	L23-242-1	Allentown/France	Positive
15	MR_Legionella_10	L14-319	SG5 Dallas	Negative
16	MR_Legionella_14	L14-233	OLDA	Negative
17	MR_Legionella_15	L15-118	OLDA	Negative
18	MR_Legionella_16	DingerPL 11	Bellingham	Negative
19	MR_Legionella_19	L23-235	Camperdown	Negative
20	MR_Legionella_20	L23-229	Bellingham	Negative
21	MR_Legionella_21	L23-187	OLDA	Negative
22	MR_Legionella_22	L23-072	OLDA	Negative
23	MR_Legionella_23	L22-473-1	OLDA	Negative
24	MR_Legionella_24	L22-318	OLDA	Negative
25	MR_Legionella_25	L22-270	OLDA	Negative
26	MR_Legionella_26	L22-118-1	OLDA	Negative
27	MR_Legionella_27	L22-59	OLDA	Negative
28	MR_Legionella_28	L22-3	Bellingham	Negative
29	MR_Legionella_29	L21-392	Bellingham	Negative
30	MR_Legionella_30	L21-390	OLDA	Negative
31	MR_Legionella_31	L21-388	OLDA	Negative
32	MR_Legionella_32	L21-345	OLDA	Negative
33	MR_Legionella_33	L21-273	OLDA	Negative
34	MR_Legionella_34	L21-245	OLDA	Negative
35	MR_Legionella_35	L21-148	OLDA	Negative
36	MR_Legionella_36	L21-140	OLDA	Negative
37	MR_Legionella_37	L21-80	Bellingham	Negative
38	MR_Legionella_38	L21-64	OLDA	Negative
